# Identification of hub genes in myocardial infarction by bioinformatics and machine learning: insights into inflammation and immune regulation

**DOI:** 10.3389/fmolb.2025.1607096

**Published:** 2025-06-24

**Authors:** Juan Yang, Xiang Li, Li Ma, Jun Zhang

**Affiliations:** ^1^ Emergency Room, The Second People’s Hospital of Dazu District, Chongqing, China; ^2^ Cardiac Catheterization Lab, The Tenth People’s Hospital Affiliated to Tongji University, Shanghai, China; ^3^ Department of Cardiovascular Medicine, The Tenth People’s Hospital Affiliated to Tongji University, Shanghai, China

**Keywords:** myocardial infarction, hub genes, inflammation, immune regulation, weighted gene co-expression network analysis (WGCNA), cardiac remodeling, LASSO, drug-gene interaction

## Abstract

**Objective:**

This study aims to identify and validate key genes involved in the progression of myocardial infarction (MI) and to investigate their roles in inflammatory response, immune regulation, and myocardial remodeling. A systematic analysis will be conducted using bioinformatics and machine learning methods.

**Methods:**

Gene expression data of GSE60993, GSE61144, GSE66360 and GSE48060 from four datasets were collected from the Gene Expression Omnibus (GEO) database. Differentially expressed genes (DEGs) between MI samples and normal samples were screened by the limma package. Weighted gene co-expression network analysis (WGCNA) was employed to identify genetic modules associated with MI. Core genes in key modules were screened using LASSO regression and support vector machine recursive feature elimination (SVM-RFE). These genes were then subjected to functional enrichment analysis, including Kyoto Encyclopedia of Genes and Genomes (KEGG), Gene Ontology (GO), and Gene Set Enrichment Analysis (GSEA). The CIBERSORT algorithm was utilized to evaluate immune cell infiltration patterns. Finally, potential therapeutic targets were explored through drug-gene interaction analysis using the DGIdb database.

**Results:**

After correcting for batch effects across datasets, we identified 687 differentially expressed genes (DEGs), including 405 upregulated and 282 downregulated genes. WGCNA analysis identified a highly correlated module with MI (turquoise module) containing 324 genes. Integrative machine learning (LASSO regression and SVM-RFE) and validation identified five key MI-associated genes: ANPEP, S100A9, MMP9, DAPK2, and FCAR. These genes were functionally enriched in inflammatory and immune-related pathways and correlated with immune cell infiltration, particularly neutrophils and macrophages. Notably, S100A9, FCAR, and MMP9 emerged as druggable targets.

**Conclusion:**

The five hub genes identified in this study (ANPEP, S100A9, MMP9, DAPK2, and FCAR) significantly contribute to MI development by modulating inflammatory responses and immune regulation. Their strong association with MI pathogenesis highlights their potential as diagnostic markers and therapeutic targets, which may lead to new clinical applications for MI management.

## Introduction

Myocardial Infarction (MI) is one of the most common cardiovascular diseases worldwide (You et al., 2023; Reed et al., 2017). Its pathogenesis is usually triggered by acute occlusion of coronary arteries, resulting in prolonged local myocardial ischemia and hypoxia, which leads to irreversible damage and necrosis of cardiomyocytes, followed by triggers of a series of complex molecular and cellular responses, such as inflammatory response, immune regulation, apoptosis, and myocardial remodeling (Oliveira et al., 2018; Ramos-Regalado et al., 2024; Liu G. et al., 2024; Kim et al., 2024; Yuan et al., 2024). The inflammatory response is one of the earliest and most important physiologic responses after MI, both contributing to tissue repair but also potentially exacerbating myocardial injury. Subsequent immune modulation and apoptotic processes further affect cardiac function recovery after myocardial injury, which in turn leads to the development of cardiac insufficiency and chronic heart failure (Zhang et al., 2022; Mahtta et al., 2020; Prabhu and Frangogiannis, 2016).

Although early diagnostic and therapeutic tools for MI have progressed over the past decades, the underlying molecular mechanisms have not been fully elucidated. This knowledge gap continues to hinder the development of precise diagnostic and therapeutic strategies (Berezin and Berezin, 2020). Consequently, identifying key MI-associated genes and their molecular pathways will deepen our understanding of disease pathogenesis while revealing potential targets for personalized therapeutic development.

In recent years, the widespread application of high-throughput sequencing technologies and advanced bioinformatics approaches has enabled increasing research focus on gene expression alterations in myocardial infarction and their functional roles in disease pathogenesis (Mujalli et al., 2020; Li et al., 2019). The identification and functional analysis of differentially expressed genes (DEGs) have been widely used to explore the molecular mechanisms associated with MI (Tian et al., 2023; Wang and Dou, 2024). A study has showed that ten core genes (Timp1, Sparc, Spp1, etc.) are directly involved in the process of myocardial fibrosis and ventricular remodeling after MI by regulating extracellular matrix remodeling and macrophage-driven inflammatory responses (Wang et al., 2022). Core genes such as ADPN promote M2 macrophage polarization, reduce inflammatory responses, and enhance lymphangiogenesis by regulation of the IL6/ADPN/HMGB1 axis, thereby alleviating myocardial injury and improving clinical prognosis after MI (Zheng et al., 2024). A comprehensive dissection of MI pathogenesis, with particular emphasis on three pivotal biological processes (inflammatory activation, immune modulation, and fibrotic transformation), is clinically imperative for advancing precision cardiology.

In addition to inflammatory factors, matrix metalloproteinases (MMPs) exert a pivotal role in myocardial remodeling after MI (You et al., 2023; Mortezapour et al., 2023). MMPs promote myocardial fibrosis and scarring by degrading the extracellular matrix, which protects the heart from further damage to a certain extent but may lead to cardiac insufficiency in the long term (Li and Feldman, 2001; Frangogiannis, 2017). MMP9, whose expression is significantly upregulated after MI, is thought to be closely linked with ventricular remodeling and the development of heart failure (You et al., 2023). In addition, Emerging evidence has established that oxidative stress serves as a critical mediator in myocardial ischemia-reperfusion injury (I/R), where excessive reactive oxygen species (ROS) generation significantly exacerbates cardiomyocyte damage and promotes apoptotic pathways (Krebber et al., 2020; Boťanská et al., 2021). The application of antioxidants has been shown to attenuate myocardial injury after MI, which also highlights that modulation of oxidative stress pathways is one of the important strategies for the future treatment of MI (Wang and Kang, 2020; Jin and Kang, 2024). However, while genes such as S100A9 and MMP9, and pathways such as NF-κB, are known to be involved in MI, a comprehensive analysis that integrates multiple datasets and employs a multi-stage computational pipeline (including WGCNA and machine learning) to identify a robust signature of hub regulatory genes and systematically characterize their collective association with specific immune cell infiltrates and druggable targets in MI remains a key area for advancement (Marinković et al., 2020; Yang et al., 2024; Liu et al., 2020; Zhuang et al., 2023).

In this study, we performed an in-depth bioinformatics analysis using publicly available gene expression microarray data from MI patients and normal controls. First, DEGs between MI patients and normal population were identified through multi-omics data integration. Next, we performed Gene Ontology (GO) enrichment and Kyoto Encyclopedia of Genes and Genomes (KEGG) pathway enrichment analyses of these differential genes, revealing the roles of these genes in key BP such as inflammatory response, immune regulation and myocardial fibrosis. In addition, the core genes (hub genes) were screened by weighted gene co-expression network analysis (WGCNA) combined with machine learning, and the functions of these core genes were further elucidated by construction of protein interaction networks (PPIs). We also behaved immune infiltration analysis of the core genes to explore their expression patterns in different immune cells. Finally, through drug-gene interaction analysis, we identified potential therapeutic targets, rendering new biological basis and strategies for precision diagnosis and personalized treatment of MI.

## Data and methods

### Data collection and analysis

The gene expression datasets analyzed in this study were retrieved from the Gene Expression Omnibus (GEO) database (https://www.ncbi.nlm.nih.gov/geo/), including four datasets, GSE60993, GSE61144, GSE66360 and GSE48060. Among them, the GSE60993, GSE61144 and GSE66360 datasets were used for differential expression analysis, while the GSE48060 served as an independent validation set for the validation of the analyzed results of the reliability. The specific information of each dataset was shown in Table 1. GSE60993 (Platform: GPL6884; Organism: *Homo sapiens*) provided peripheral blood samples from 7 normal controls and 26 patients with acute coronary syndrome (ACS). For the purposes of this study, ACS patients, encompassing ST-elevation myocardial infarction (STEMI), Non-ST-elevation MI (NSTEMI), and unstable angina (UA), all presenting within 4 h of chest pain onset, were collectively categorized as the MI group. And the GSE61144 dataset (Platform: GPL6106; Organism: *Homo sapiens*) contributed peripheral blood samples; 7 normal control samples and 10 MI samples were included from this dataset for our analysis. GSE66360 (Platform: GPL570; Organism: *Homo sapiens*) involved the analysis of CD146+ circulating endothelial cells (CECs) isolated from 50 healthy individuals (normal controls) and 49 patients with acute myocardial infarction (MI samples). The validation dataset, GSE48060 (Platform: GPL570; Organism: *Homo sapiens*), was reserved as an independent external validation cohort. This dataset consisted of peripheral blood samples from 21 control subjects and 31 MI patients.

**TABLE 1:** Details of the GEO datasets included in the current study.

Dataset	Samples (normal VS MI samples)	Platform	Tissue	Group
GSE60993	7 VS 26	GPL6884	Blood	Training set
GSE61144	7 VS 10	GPL6106	Blood	Training set
GSE66360	50 VS 49	GPL570	CD146+ Circulating endothelial cells	Training set
GSE48060	21 VS 31	GPL570	Blood	Validation set

Gene expression data for each dataset (GSE60993, GSE61144, GSE66360, and GSE48060) were retrieved from the Gene Expression Omnibus (GEO) public database, typically as series matrix files. Initial preprocessing steps were applied consistently to each dataset. Probe identifiers were mapped to official gene symbols using the corresponding platform annotation files (e.g., GPL6884 for GSE60993, GPL6106 for GSE61144, GPL570 for GSE66360 and GSE48060). In instances where a single probe ID mapped to multiple gene symbols (often separated by “///” in annotation files), the first gene symbol listed was retained for that probe. Following probe-to-gene mapping, multiple probes could correspond to the same gene symbol. To obtain a single expression value per gene, the expression values of probes mapping to the same gene symbol were summarized by calculating their average using the avereps function within the limma R package. Genes with no corresponding gene symbol after annotation were removed.

To mitigate non-biological experimental variations in the integrated training cohort (derived from datasets GSE60993, GSE61144, and GSE66360), batch effects were corrected using the ComBat function from the sva R package (Version 3.52.0). This function was applied to the merged expression matrix, with the dataset of origin for each sample designated as the batch variable. The ComBat algorithm was implemented using its default parametric empirical Bayes framework, without the inclusion of a model matrix for specific biological covariates at this correction stage. The successful reduction of batch-related data clustering was confirmed by visual inspection of boxplots of the expression data before and after correction.

### Selection of DEGs

The limma package (Version 3.60.5) was applied to screen for DEGs between MI samples and Normal samples, with DEGs threshold at P < 0.01 and |log2(FC)| > 0.585. Subsequently, pheatmap (Version 1.0.12) and ggplot2 (Version 3.5.1) to were taken to plot volcano and heat maps, respectively.

### WGCNA identifies key module genes

For further exploration of the potential biological functions of DEGs, we performed WGCNA on the DEGs screened above. WGCNA is a systems biology approach widely used to analyze gene co-expression patterns among different samples (You et al., 2023). By construction of gene co-expression networks, gene modules with highly co-expressed genes could be identified with further exploration of the relationship between these modules and MI.

### Machine learning screening of hub genes in key modules

In order to screen out core genes of the key modules in WGCNA analysis, we applied LASSO regression analysis for feature selection. First, the gene expression matrix and sample classification information of the key modules of WGCNA were read as independent and dependent variables respectively, ensued with data conversion to matrix form and the dependent variables to dichotomous variables (MI vs. Normal samples). The optimal lambda value for the LASSO model was determined through 10-fold cross-validation using the cv. glmnet function, with the mean squared error (MSE) serving as the evaluation metric. Based on this parameter, we constructed the LASSO regression model and showed the trend of coefficients with lambda by regression path diagram.

The LASSO regression effectively contracted the regression coefficients by L1 regularization and screened out the core genes with significant contributions. We identified the core genes by selecting those with non-zero coefficients under the optimal lambda value in LASSO regression.

To assess the reliability of the core gene screening, we further screened the core genes screened by LASSO using the SVM-RFE method. SVM-RFE performs recursive feature elimination of the genes by five-fold cross-validation (k = 5), which leads to the screening of key gene features with high classification performance.

For exploration of the interactions and associations of hub genes with other genes, gene association networks were constructed via the GeneMANIA database. This database integrates a wide range of functional association information, including physical interactions, co-expression, and co-localization, which can comprehensively predict the functions and interrelationships of genes (Warde-Farley et al., 2010).

### Consensus cluster analysis

Using consensus clustering analysis, we identified molecular subtypes associated with the core genes screened. We used the “ConsensusClusterPlus (1.58.0)” R package for consensus clustering analysis, which clustered samples by the k-means algorithm and evaluated the robustness of different numbers of clusters. The optimal cluster count was assessed through 1,000 subsampling replicates (80% samples per iteration, pItem = 0.8) with maxK = 9. Euclidean distance was chosen as the distance calculation method for clustering.

Through consensus clustering, we generated consensus clustering heat maps, consensus cumulative distribution function (CDF) maps, and incremental area maps. Sample Inconsistency Consistency (IC) and Cluster Consistency (CLC) were calculated for each possible number of clusters (k-value). Finally, based on the analysis results, we selected the optimal number of clusters and classified the samples.

### Functional enrichment analysis

With the aim of assessing biological functions of the key genes, the screened DEGs were subjected to GO functional enrichment analysis and pathway enrichment analysis of KEGG by clusterProfiler (Version 4.12.6) R package. For GO enrichment analysis, we evaluated the enrichment of genes in three major categories: BP, molecular function (MF), and cellular component (CC) with a significance threshold of P < 0.05 for statistically enriched terms.

### Gene Set Enrichment Analysis (GSEA) enrichment analysis

To explore the functional enrichment of differentially expressed genes (DEGs), we conducted Gene Set Enrichment Analysis (GSEA). This method evaluates whether a predefined gene set shows statistically significant enrichment in a ranked gene list, revealing potential associations with specific biological pathways. The analysis was performed using the clusterProfiler R package (v4.12.6), with the MSigDB Hallmark gene sets as the reference. A significance threshold of P < 0.05 was applied to identify enriched pathways.

### Immune infiltration analysis

To assess immune cell composition, we performed immune infiltration analysis using the CIBERSORT algorithm, which quantifies the relative proportions of 22 immune cell types in the samples. The differences in immune cell infiltration across groups were visualized using box plots. Furthermore, we identified five key genes (ANPEP, S100A9, MMP9, DAPK2, and FCAR) and evaluated their correlations with immune cells using Pearson’s method. The resulting associations were visualized as a correlation heatmap generated with the corrplot R package.

### Drug-gene interaction analysis

To explore the potential drug targets of hub genes, the screened hub genes were analyzed for drug-gene interactions via the Drug-Gene Interaction Database (DGIdb, https://www.dgidb.org/).

### Statistical analysis

All statistical analyses were conducted using R software (v4.4.1). The diagnostic performance of key genes was evaluated using Receiver Operating Characteristic (ROC) curves, generated with the pROC package (v1.18.5). The Area Under the Curve (AUC) was computed for each gene, with P < 0.05 considered statistically significant.

## Results

### Identification of DEGs in MI

**FIGURE 1:**
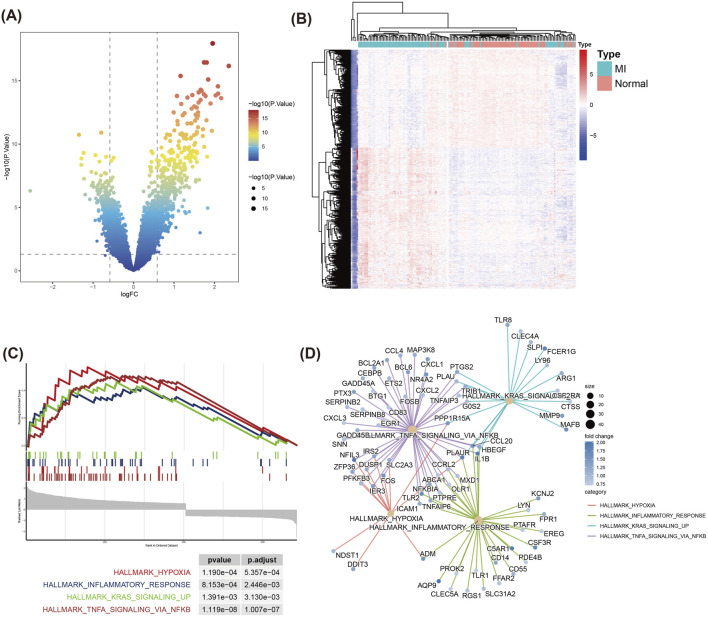
Differential gene expression and GSEA enrichment analysis in MI and Normal samples. **(A)** Volcano plot displays the distribution of DEGs between MI and Normal groups. The DEGs were filtered using |log2 Fold Change| > 0.585 and P < 0.05 as the thresholds. Red and green dots represent significantly upregulated and downregulated genes respectively. **(B)** Heatmap of the top DEGs between MI and Normal samples. Rows represent genes, and columns represent samples. Red indicates upregulation, while blue denotes downregulation. **(C)** GSEA of DEGs. **(D)** Pathway-gene interaction network from GSEA results. Circle sizes represent fold changes, and color intensity reflects statistical significance (*P*-value).

Following batch effect correction across datasets, we conducted differential expression analysis between myocardial infarction (MI) and normal samples (Supplementary Figure S1). Using the limma package, we identified 687 differentially expressed genes (DEGs) with thresholds of |log_2_FC| > 0.585 and P < 0.05, including 405 upregulated and 282 downregulated genes (Figures 1A,B). To elucidate their biological roles, GO and KEGG enrichment analyses were performed. GO terms revealed significant enrichment in (1) Positive regulation of cytokine production, (Reed et al., 2017), Immune response-activating signaling pathway, (Oliveira et al., 2018), Immune receptor activity (Supplementary Figure S2A). In addition, KEGG analysis exhibited obvious enrichment in the Cytokine-cytokine receptor interaction, NF-kappa B signaling and Ribosome pathways (Supplementary Figure S2B).

In addition, we used GSEA to assess the enrichment of DEGs in known functional pathways. GSEA analysis displayed that MI-related pathways, including TNFA signaling via NFKB, Hypoxia, Inflammatory response, and KRAS signaling_up, were prominently enriched in the MI group (Figure 1C). The pathway-gene interaction network demonstrated that critical genes—including CXCL1, PTGS2, and NFKBIA—were prominently clustered within the TNFA/NFKB signaling pathway (Figure 1D). This finding suggests these genes may serve as central regulators in the inflammatory and immune mechanisms underlying MI. Hypoxia and Inflammatory response pathways also contained multiple genes related to immune response and stress response-related genes, such as ADM and IER3.

### WGCNA key module identification

**FIGURE 2:**
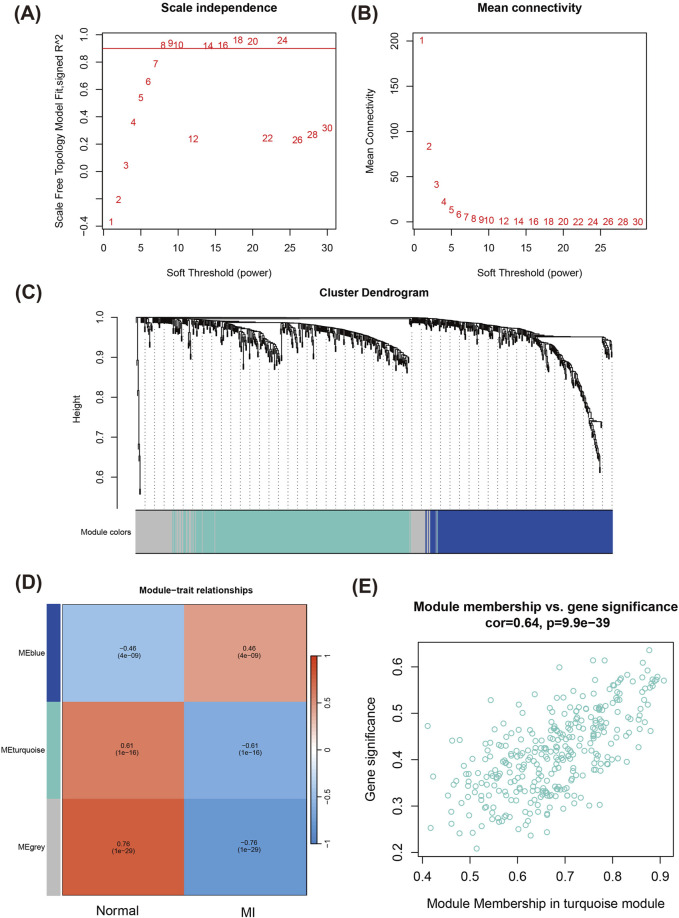
WGCNA for identifying key modules related to MI. **(A)** Scale-free topology fit index as a function of the soft-thresholding power. **(B)** Mean connectivity analysis across different soft-thresholding powers. **(C)** Cluster dendrogram of genes, with modules indicated by different colors. Each branch represents a gene, and similar genes are grouped into modules. **(D)** Heatmap of module-trait relationships registering the correlation between different gene modules and MI phenotype. **(E)** Scatter plot of module membership vs. gene significance in the turquoise module.

To systematically investigate the biological roles of DEGs, we performed WGCNA to identify key modules associated with MI. First, by analysis of the fit of different soft thresholds to the scale-free network (Figure 2A) and the average connectivity between genes, the most suitable soft threshold of 8 was identified to ensure that the network had scale-free properties and maintained high gene connectivity (Figures 2A,B). Next, we constructed gene co-expression networks and enabled clustering analysis of gene modules (Figure 2C). The heatmap of module-trait relationships revealed that the turquoise module demonstrated the strongest association with the MI group (correlation coefficient R = 0.61, P = 1 × 1e-16), indicating that genes within this module likely play a critical role in MI pathogenesis (Figure 2D).

Further analysis of the correlation between Module Membership (MM) and Gene Significance (GS) of the genes in the turquoise module showed a strong positive correlation (R = 0.64, P = 9.9e-39) (Figure 2E), suggesting that the module of genes both occupy important positions in the network and also have significant functions in the BP of MI.

**FIGURE 3:**
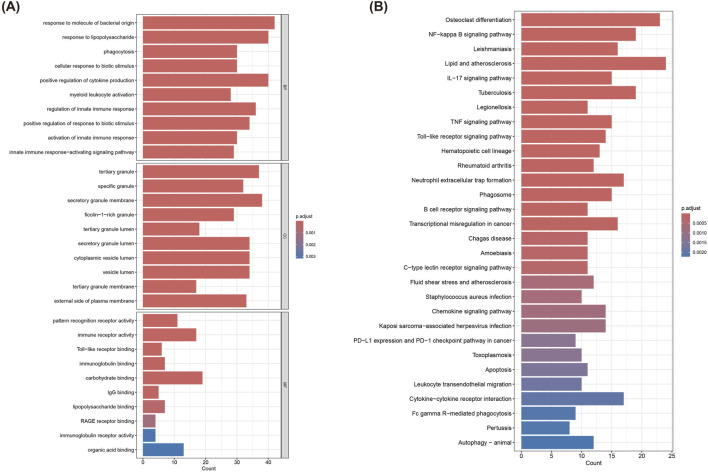
Functional enrichment analysis of genes in the turquoise module. **(A)** GO enrichment analysis of 324 genes in the turquoise module. **(B)** KEGG pathway analysis of 324 genes in the turquoise module.

GO and KEGG analyses were applied to the 324 turquoise module genes to decipher their potential roles in MI-related pathways and functions. The GO enrichment results (Figure 3A) revealed significant enrichment in immune response and innate immunity activation-related biological processes, including (You et al., 2023) Response to molecule of bacterial origin, (Reed et al., 2017), Phagocytosis, (Oliveira et al., 2018), Positive regulation of cytokine production (Ramos-Regalado et al., 2024) Myeloid leukocyte activation and so on. Meanwhile, in terms of CC, these genes were enriched in organelles such as tertiary granule, secretory granule membrane, and cytoplasmic vesicle lumen. Besides, when it comes to MF, they were significantly enriched in pattern recognition receptor activity, immune receptor activity, and organic acid binding, etc. KEGG pathway analysis showed significant enrichment of these pathways (Figure 3B): NF-kappa B signaling pathway, TNF signaling pathway, IL-17 signaling pathway, B cell receptor signaling pathway, C-type lectin receptor signaling pathway, Lipid and atherosclerosis, and Phagosome, etc. These pathways were closely related to immune responses, inflammatory responses, and pathological processes associated with cardiovascular diseases, rendering important clues to further understand the molecular mechanisms of these genes in MI.

### Machine learning identifies hub genes

**FIGURE 4:**
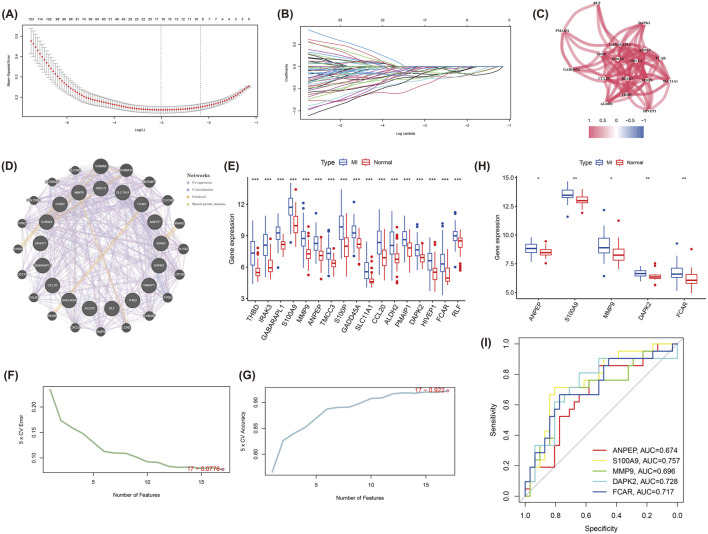
LASSO regression and SVM-RFE analysis to identify hub genes in the turquoise module. **(A)** The LASSO regression model used to identify core genes from the 324 genes in the turquoise module. **(B)** LASSO coefficient profiles for each gene as the regularization strength (lambda) varies. **(C)** Correlation network of the 17 selected genes from LASSO. **(D)** GeneMANIA network of the 17 hub genes and 20 related genes, displaying interactions based on co-expression, co-localization, predictions, and shared protein domains. **(E)** Boxplot analysis of the 17 genes’ expression levels in MI and normal groups, revealing significant differences between the two groups. **(F)** The cross-validation error curve from the SVM-RFE analysis, registering the optimal number of features for classification. **(G)** The accuracy curve from SVM-RFE analysis, indicating the number of features providing the best classification performance. **(H)** Boxplots of the five hub genes (ANPEP, S100A9, MMP9, DAPK2, and FCAR) showing significant differences in expression between MI and normal samples in the validation dataset (GSE48060). **(I)** ROC for the five hub genes, demonstrating their diagnostic ability in distinguishing MI from normal samples.

In order to screen for core genes significantly associated with MI in the turquoise module, we behaved LASSO regression analyses on the 324 genes included in this module. These mentioned genes were screened for subsequent analysis by selection of the optimal lambda values during cross-validation (Figures 4A,B). Results of the correlation network map of the 17 genes screened showed strong interactions between multiple core genes, such as a stronger association between ANPEP, MMP9, S100A9, PMAIP1, DAPK2 and FCAR (Figure 4C). Subsequently, an interaction network of these 17 core genes with 20 related genes was constructed via GeneMANIA, displaying the main involvement in Co-expression, Co-localization, Predicted and Shared protein domains (Figure 4D). Next, Subsequent analysis demonstrated significant differential expression of these candidate genes between MI patients and normal group (Figure 4E). In particular, the genes further screened by SVM-RFE exhibted high classification performance with results of SVM analysis were demonstrated in Figures 4F,G.

In the validation set (GSE48060), we identified expression levels of five genes (ANPEP, S100A9, MMP9 and DAPK2) were equally significant between the two groups (Figure 4H), which were identified as hub genes with their diagnostic performance evaluated by ROC curve analysis. The results indicated that these genes demonstrated a moderate ability to distinguish MI samples from normal samples. The corresponding Area Under the Curve (AUC) values were ANPEP: 0.674, S100A9: 0.757, MMP9: 0.696, DAPK2: 0.728, and FCAR: 0.717, respectively (Figure 4I).

### Cointegration analysis

**FIGURE 5:**
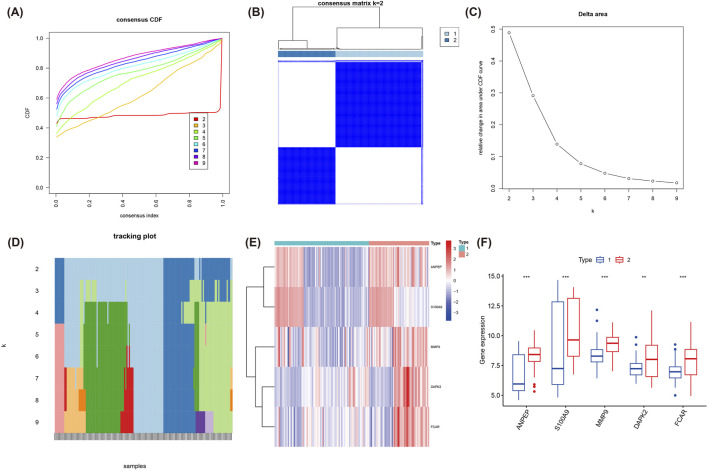
Consensus clustering analysis based on the expression of the five hub genes. **(A)** Consensus CDF plot for k = 2 to 9 clusters, denoting the CDF across consensus indices. **(B)** Consensus matrix for k = 2, presenting the stable and distinct clusters identified. **(C)** Delta area plot, indicating the relative change in area under the CDF curve for each k value. **(D)** Tracking plot showing the cluster stability across different values of k. **(E)** Heatmap of the expression of the five hub genes (ANPEP, S100A9, MMP9, DAPK2, FCAR) across the two identified clusters. **(F)** Boxplot illustrating the significant differences in the expression of the five hub genes between cluster 1 and cluster 2.

Using the expression patterns of five hub genes, we performed consensus clustering analysis, which identified two distinct molecular clusters with significantly differential gene expression profiles (Figures 5A–D). Heatmaps and box plots showed expressive differences between cluster 1 and cluster 2 (Figures 5E,F). Among them, expression levels of ANPEP, S100A9, MMP9, DAPK2, and FCAR were prominently higher in cluster 2. Overall, expression levels of these five hub genes were usually higher in cluster 2 than in cluster 1, providing new insights into the molecular typing of MI and potentially informing personalized therapy in the future.

### Immune infiltration analysis

**FIGURE 6:**
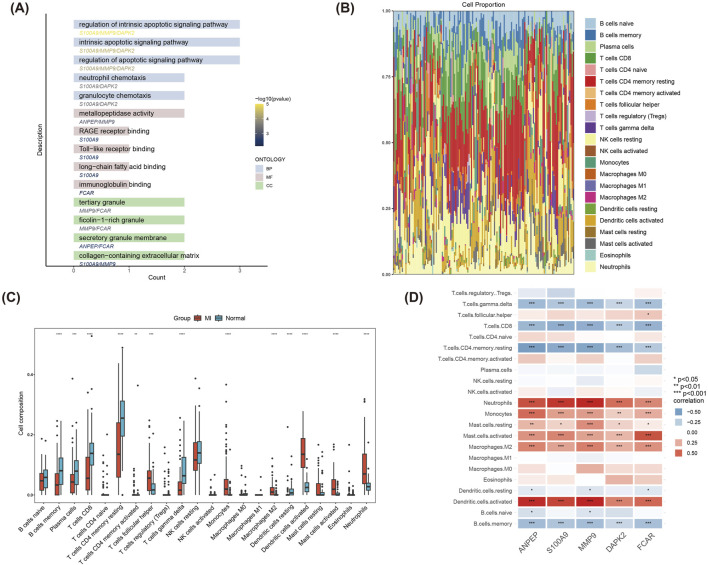
GO enrichment and immune infiltration analysis of hub genes. **(A)** GO functional enrichment analysis of the five hub genes (ANPEP, S100A9, MMP9, DAPK2, FCAR). **(B)** Stacked bar plot depicting the composition of 22 immune cell types in MI and Normal groups, highlighting significant differences in immune cell proportions. **(C)** Boxplot comparing immune cell infiltration levels between MI and Normal groups. **(D)** Correlation heatmap of the five hub genes with immune cells.

Functional enrichment analysis of the five hub genes (ANPEP, S100A9, MMP9, DAPK2, FCAR) revealed their significant involvement in BP related to regulation of intrinsic apoptotic signaling pathway, neutrophil chemotaxis, Toll-like receptor binding and so on (Figure 6A). Such mentioned biological functions are closely linked with inflammatory and immune response mechanisms of MI.

The immune infiltration analysis demonstrated significant differences (P < 0.05) in the compositional distribution of 22 immune cell types between the MI and control groups (Figure 6B). Results of box plot registered that the infiltration of immune cells such as Dendritic cells activated, Neutrophils, Mast cells activated and T cells follicular helper was significantly increased in the MI group (Figure 6C). As shown in Figure 6D, correlations between the five hub genes and immune cells were further analyzed. Hub genes were significantly positively correlated with Neutrophils, Monocytes, and Dendritic. cells.activated, and significantly positively correlated with T. cells.gamma.delta, T. cells.CD4. memory.resting and B. cells.memory significantly negatively correlated. These findings revealed the potential role of hub genes in immune regulation and provide new clues for understanding immune mechanisms in MI.

### Drug-gene interaction analysis to screen potential drugs

Through drug-gene interaction analysis, we screened a number of potential drugs associated with S100A9, FCAR and MMP9. According to results in Table 2, the S100A9 gene registered high interaction scores with two drugs, namely, TASQUINIMOD and PAQUINIMOD (Interaction Score = 26.25384), suggesting that these drugs might be potential targets for S100A9.

**TABLE 2:** Potential drug-gene interactions based on DGIdb analysis (Interaction Score >2.0).

Gene	Drug	Interaction score
S100A9	TASQUINIMOD	26.25384
S100A9	PAQUINIMOD	26.25384
FCAR	SK1-I	4.375639
FCAR	COMPOUND 28	4.375639
MMP9	CARBOXYLATED GLUCOSAMINE	4.200614
MMP9	ANDECALIXIMAB	4.200614
FCAR	COMPOUND 59	2.18782
FCAR	SLM6071469	2.18782
FCAR	MP-A08	2.18782
FCAR	COMPOUND 49	2.18782
FCAR	COMPOUND 27D	2.18782
FCAR	SLC4101431	2.18782
FCAR	PF-543	2.18782
FCAR	COMPOUND 55	2.18782
FCAR	COMPOUND 60	2.18782
MMP9	DP-B99	2.100307

As for the FCAR gene, SK1-I and COMPOUND 28 presented the highest interaction score (Interaction Score = 4.375639), meanwhile, the interaction CARBOXYLATED GLUCOSAMINE and ANDECALIXIMAB with MMP9 (Interaction Score = 4.200614), indicating that these drugs may play a role in the treatment of MI by targeting FCAR and MMP9 genes.

## Discussion

MI is one of the leading causes of death worldwide, placing a huge burden on life quality of patients and socioeconomic status. An in-depth understanding of its pathogenesis makes contributions to developing effective preventive and therapeutic strategies. While some of the identified hub genes, such as S100A9 and MMP9, and their associated pathways like NF-κB and TNF signaling, are individually well-documented in MI pathogenesis, the distinct contribution of this study lies in its integrative multi-dataset approach and machine learning-based prioritization. By systematically analyzing four GEO datasets and employing a pipeline encompassing WGCNA to identify clinically relevant modules followed by LASSO and SVM-RFE for robust feature selection, we have identified a specific five-gene signature (ANPEP, S100A9, FCAR, MMP9, and DAPK2) as centrally implicated. Our subsequent analysis provides novel insights into the collective association of this signature with specific immune cell infiltration patterns and highlights potential targeted therapeutic avenues based on these prioritised genes. ROC analysis of these 5 core genes performed that these genes possessed good diagnostic efficacy in patients with MI with high AUC values, which further confirmed their potential diagnostic value in MI.

Furthermore, through GO enrichment analysis, we investigated the biological functions and pathways of these hub genes. The results revealed their significant enrichment in inflammatory response, immune regulation, apoptosis, and extracellular matrix remodeling – all pathways critically involved in the pathological progression of myocardial infarction. Inflammatory response and immune regulation after MI leverage crucial roles in the development and progression of the disease. During the acute phase of MI, cardiomyocyte necrosis and hypoxia trigger the massive release of damage-associated molecular patterns (DAMPs), which potently activate the innate immune system, especially the recruitment of neutrophils and macrophages (You et al., 2023). By secretion of cytokines and chemokines, these immune cells contribute to the expansion of the inflammatory response and play a dual role in the process of tissue repair: on the one hand, they contribute to the removal of necrotic tissue, but on the other hand, they may also lead to cardiac fibrosis and dysfunction through hyperactivation (Frangogiannis, 2014). Enrichment analysis paraded significant enrichment of several inflammation- and immune-related signaling pathways, such as the NF-κB signaling pathway and the TNF signaling pathway, which are thought to exert an significant role in inflammatory expansion and tissue repair after MI (Martí-Carvajal et al., 2024; Dong et al., 2024).

The immune infiltration profiling further elucidated the pivotal functions of key immune cells - particularly neutrophils and macrophages - in myocardial infarction progression: neutrophils and macrophages. Neutrophils are one of the first cells to infiltrate in the early stages of MI, and they promote the inflammatory response and tissue damage by releasing ROS and protein hydrolases (Ma et al., 2013). As the inflammatory response progresses, macrophages play a key role in the differentiation and repair process in the late stage of MI (Tugal et al., 2013). Early M1-type macrophages mainly promote inflammatory responses, whereas late M2-type macrophages facilitate tissue repair and fibrosis (Shiraishi et al., 2016; Wynn and Vannella, 2016). Therefore, modulation of neutrophil and macrophage activity may become an important therapeutic target to attenuate inflammatory injury and promote cardiac function recovery after MI.

ANPEP is an aminopeptidase that can participate in the processing of various peptides, including angiotensin III and IV, and plays an important role in angiogenesis (Rangel et al., 2007; Vaswani et al., 2015). ANPEP expression is upregulated in the plasma of patients with nonalcoholic fatty liver disease (NAFLD) and cirrhosis, suggesting that it may be involved in the progression of NAFLD and may serve as a potential drug target (Niu et al., 2019). However, ANPEP expression is downregulated in sacubitril/valsartan-resistant (SVR) patients with heart failure, indicating that it may be involved in the development of SVR and serve as a potential therapeutic target or biomarker (Su et al., 2023). FCAR encodes a transmembrane glycoprotein receptor in the Fc region of IgA that is highly expressed in neutrophils (You et al., 2023), which is consistent with the results of our immune infiltration analysis: FCAR was significantly associated with neutrophils, confirming the important role of FCAR in neutrophils. In addition, FCAR gene expression levels were significantly elevated in patients with sepsis, which may play as a potential biomarker for the diagnosis of sepsis and be involved in the formation of neutrophil extracellular traps (NETs) (You et al., 2023; Zhang and Zhang, 2024). Notably, the function of receptors in immune cells, such as FCAR, may be significantly modulated by their localization within specialized membrane microdomains known as lipid rafts. These lipid rafts serve as critical signaling platforms in immune cells, concentrating receptors and downstream signaling molecules, thereby influencing processes such as vascular inflammation and thrombosis, as comprehensively reviewed in the context of diseases involving autoantibodies (Capozzi et al., 2023). Given the pivotal role of neutrophils in MI and the high expression of FCAR on these cells, FCAR-mediated signaling during MI may depend on its association with lipid rafts, potentially impacting neutrophil activation and the formation of NETs—the latter being recognized contributors to MI pathology. Future studies could explore the role of FCAR localization in lipid rafts within neutrophils during MI.

S100 family proteins, especially S100A9, have been shown to exert a key role in inflammatory regulation and disease progression (Shabani et al., 2018). In a variety of tumors, S100A9 forms a heterodimer with S100A8, S100A8/A9, which interferes with the tumor microenvironment and metabolism, promotes tumorigenesis, progression and metastasis, and serves as a tumor diagnostic and prognostic marker as well as a potential therapeutic target (Wang et al., 2018; Ye et al., 2019; Chung et al., 2023). Furthermore, in hepatocellular carcinoma, S100A9 accelerates tumor progression by recruiting ubiquitin specific peptidase 10 (USP10) and Phosphoglycerate Mutase Family Member 5 (PGMFM5) to form a trimer that facilitates mitochondrial fission and ROS production (Zhong et al., 2022). In obesity-related diseases, S100A9 activates the NFκB signaling pathway by binding to TLR4, inhibiting the differentiation of M2-like macrophages and enhancing pro-inflammatory functions, bring about increased inflammation and impaired wound repair (Franz et al., 2022). The pro-inflammatory signaling cascades initiated by S100A9, particularly its engagement with receptors such as Toll-like Receptor 4 (TLR4), may also be spatially regulated by lipid rafts. These membrane microdomains are considered organizing centers for receptor signaling in immune cells, playing a role in vascular inflammation and thrombosis (Capozzi et al., 2023). The review by Capozzi et al. highlights that TLR4 signaling can occur within lipid rafts, suggesting that during MI, the interaction of S100A9 with TLR4 on macrophages and neutrophils might be modulated by the integrity and composition of these rafts. This organization could be critical for the full activation of downstream pathways, such as NF-κB, thereby influencing the intensity of the inflammatory response and subsequent cardiac remodeling post-MI. Investigating the dependence of S100A9-TLR4 signaling on lipid raft integrity in the context of MI could offer new therapeutic avenues. Similarly, in sepsis-induced acute liver injury, S100A9 exacerbates liver dysfunction and injury by regulation of AKT-AMPK-dependent mitochondrial energy metabolism (Zhang et al., 2023). The above studies further demonstrate the proinflammatory role of S100A9 in multiple organs, revealing its pathogenic role in inflammation and related diseases. In heart-related diseases, S100A9 also plays a crucial role in post-MI inflammation and repair. It has been discovered that S100A9 hi macrophages promote the acute inflammatory response and fibrotic process after MI by activation of Myd88/NFκB/NLRP3 and Tgf-β/p-smad3 signaling pathways (Marinković et al., 2020; Shen S. et al., 2024). In addition, S100A9 inhibits Atg9a transcription via prevention of its entry into the nucleus through binding to HIF-1α, which in turn inhibits autophagy and leads to cardiac dysfunction (Zhi et al., 2023). Our immune infiltration analysis provides further mechanistic insight, revealing a significant positive correlation between S100A9 expression and the infiltration of neutrophils, monocytes, and activated dendritic cells within the MI microenvironment. This specific association highlights S100A9 as a key mediator orchestrating the complex interplay of these pro-inflammatory myeloid cells during acute myocardial injury and the subsequent repair phase. Such insights into the specific immune context are critical for guiding therapeutic strategies. The identification herein of TASQUINIMOD and PAQUINIMOD as high-confidence interacting drugs for S100A9 (Interaction Score = 26.25) offers a particularly promising avenue. These agents, recognized for immunomodulatory effects that include S100A9 targeting, could potentially mitigate MI-induced inflammation (Jin et al., 2022; Talley et al., 2021). Specifically, they may act by attenuating S100A9-driven activation and recruitment of these key myeloid cells. This potential mechanism, underscored by our integrative analysis which links S100A9’s specific immune correlations with its druggability, warrants further investigation in the specific context of MI. All these findings render a theoretical basis for future therapeutic strategies targeting S100A9, which are expected to improve the prognosis of MI patients.

MMP9 exacerbates atherosclerosis, myocardial fibrosis, cardiac injury by extracellular matrix degrade and autophagy inhibition, as well as plays a pivotal role in the progression of various diseases, serving as an important target for the treatment of cardiovascular disease (Bassiouni et al., 2021; Zhang, 2022). During the onset and progression of MI, MMP9 gene polymorphisms and expression regulation have an important impact on the disease process. It has been revealed that the MMP9 rs3918242 polymorphism is associated with atherosclerotic plaque rupture and significantly increases the risk of MI (Rodríguez-Pérez et al., 2016). Besides, MMP9 exerts a key role in cardiac remodeling after MI with its downregulation of expression thought to be able to improve cardiac contractile function and attenuate cardiac damage (Goerg et al., 2021). Further, MMP9 not only plays an important role in the acute phase of MI, but also exacerbates the progression of chronic heart failure by inhibiting autophagic flux. Inhibition of MMP9 is able to inhibit the mTOR pathway by activating AMPKα, Beclin-1, and Atg7 pathways, thereby increasing autophagic flux, which in turn improves cardiac remodeling and enhances cardio-protection (Nandi et al., 2020). MMP9 exerts an important impact on atherosclerotic plaque formation and vascular remodeling, contributing to plaque rupture and exacerbating inflammatory responses with its down-regulation being effective in attenuating the progression of atherosclerosis and reducing the risk of cardiovascular disease (Ma et al., 2023). In our study, the expression level of MMP9 was significantly increased in the MI group, further validating its critical role in the occurrence and progression of MI, which offered potential clinical applications for therapeutic strategies targeting MMP9 and might open new avenues for intervention and treatment of cardiovascular diseases. Besides, this pro-inflammatory and matrix-remodeling role of MMP9 may intersect with other enzymatic systems implicated in vascular injury and thrombosis, such as heparanase. Heparanase, an endo-β-D-glucuronidase, is increasingly recognized as a critical modulator of endothelial dysfunction, inflammation, and thrombotic processes through its capacity to cleave heparan sulfate proteoglycans, thereby releasing bioactive molecules and altering the extracellular matrix and cell surface properties (Vlodavsky et al., 2021). A previous study demonstrated that pharmacological inhibition of heparanase significantly reduced tissue factor (TF) expression in both platelets and endothelial cells, key players in coagulation activation (Capozzi et al., 2021). Given that MMP9 contributes to vascular remodeling, plaque instability, and inflammation—processes often culminating in thrombotic events initiated by TF exposure—a potential mechanistic link with heparanase is conceivable in MI. For instance, MMP9-mediated degradation of the ECM could expose heparan sulfate for heparanase action or vice-versa, synergistically promoting a pro-inflammatory and procoagulant microenvironment. Both enzymes can influence endothelial activation and leukocyte trafficking (Bräuninger et al., 2023). Thus, the combined activities of MMP9 and heparanase might cooperatively drive MI pathogenesis by amplifying ECM degradation, inflammation, and the thrombotic cascade. Future research could explore this potential enzymatic crosstalk.

Furthermore, our study provides novel insights into MMP9’s immunomodulatory context in MI by demonstrating its strong positive correlation with the infiltration levels of neutrophils, monocytes, and activated dendritic cells. This suggests that MMP9 expression is closely linked to the heightened activity of these crucial immune cells involved in MI-associated inflammation and subsequent tissue remodeling. The potential therapeutic relevance of MMP9 in this specific immune setting is underscored by our drug-gene interaction analysis, which identified CARBOXYLATED GLUCOSAMINE and ANDECALIXIMAB (an anti-MMP9 antibody) as interacting drugs (Mortezapour et al., 2023; Allen et al., 2022). Targeting MMP9 with such agents could therefore not only modulate ECM degradation but also distinctly impact the MMP9-dependent functions of these key infiltrating immune cells, offering a refined strategy for mitigating adverse cardiac remodeling in MI. These specific drug-gene-immune cell associations represent an advancement beyond general knowledge of MMP9’s role.

DAPK2 is a Ca^2+^ -regulated serine/threonine kinase that leverages an important role in apoptosis and autophagy (Ber et al., 2015; Saberiyan et al., 2024). Existing studies have shown that DAPK2 serves as an important part in the development of a variety of diseases. In a rat model of diabetic cardiomyopathy, MIAT and circMAP3K5 upregulate DAPK2 expression through competitive binding to miR-22-3p, which in turn promotes cardiomyocyte apoptosis (Zhou et al., 2017; Shen M. et al., 2024). In ischemia/reperfusion injury (CI/R), DAPK2 is a direct target of miR-133a-3p and its expression is upregulated in cerebral ischemia/reperfusion injury, miR-133a-3p can attenuate CI/R injury by inhibiting the expression of DAPK2, which suggests that DAPK2 may be a potential target for the treatment of CI/R injury (Yang et al., 2023). In addition, altered DAPK2 gene expression serves as a potential biomarker for the diagnosis of a variety of diseases such as Parkinson’s disease, colorectal cancer, and gliomas (Bao et al., 2024; Li et al., 2024; Xu et al., 2020). This point further supports the broad regulatory function of DAPK2 in multiple pathological states. Consistent with these findings, the present study demonstrated a significant increase in DAPK2 mRNA expression levels in patients with MI with ROC analysis showed AUC = 0.728, indicating its good diagnostic value in the diagnosis of MI.

In addition, this study screened potential therapeutic agents targeting S100A9, FCAR and MMP9 by DGIdb drug-gene interaction analysis. The discovery of these drugs, particularly when viewed in conjunction with the specific immune cell correlations of their target genes highlighted in our study, provides a new potential direction for personalized treatment of MI. S100A9 participates in inflammatory response, and its targeting drugs may attenuate myocardial injury by inhibiting inflammatory pathways, potentially mediated by neutrophils, monocytes, and activated dendritic cells with which it strongly correlates (Liu J. et al., 2024). Drugs targeting FCAR may play a role in the late phase of MI by modulating the immune response, whereas drugs targeting MMP9 are expected to improve cardiac function by reducing myocardial fibrosis and potentially by modulating the activity of associated immune cells like neutrophils and macrophages (Nian et al., 2023; Gentek and Hoeffel, 2017). However, although these drugs register potential targeting effects, the feasibility and efficacy of their clinical application need to be verified though further experimental and clinical trials. Such studies of drug-gene interactions have brought new hope for the precision treatment of MI, however, how to translate these findings into clinical applications remains a future research priority.

This study also has some limitations. First, although we screened and integrated gene expression data of MI from multiple datasets, there may be some bias due to the limitation of sample size. The batch effect, although corrected by the algorithm, may still have a non-negligible impact on the analysis results. Therefore, further validation should be carried out in the future in combination with larger-scale, multicenter data from a unified platform. Second, this study lacks detailed clinical characterization information to combine clinical variables with gene expression data, which limits the application scenarios of the model. Introduction of more clinical information, especially the history and course data of individual patients, will help improve the diagnostic and predictive ability of the model. Besides, based on the mRNA level, our analyses failed to render necessary and direct reflection of protein function or its specific role in MI, and more experimental validation at the protein level is needed subsequently. Finally, although we identified potential drug targets, the effectiveness of these drugs in clinical applications still needs require further investigation to confirm.

In summary, through a systematic and integrative bioinformatics and machine learning approach applied to multiple MI datasets, this study identified and validated a robust five-hub gene signature (ANPEP, S100A9, MMP9, DAPK2, and FCAR). Beyond confirming their general involvement in MI, our work provides novel insights by specifically linking this signature to distinct immune cell infiltration patterns—particularly highlighting strong correlations with neutrophils, monocytes, and activated dendritic cells for key genes like S100A9 and MMP9. Furthermore, we have contextualized the therapeutic potential of these hub genes by identifying specific drug-gene interactions, offering a more refined understanding of how agents targeting S100A9 and MMP9 might exert their effects within the specific immune microenvironment of MI. These findings not only advance our perspective on the molecular mechanisms of MI, but also lay the foundation for future personalized treatment strategies. However, the specific regulatory mechanisms of the core genes and clinical effects of drug actions still need further verification in the future.

## Data Availability

The original contributions presented in the study are included in the article/Supplementary Material, further inquiries can be directed to the corresponding authors.

## Supplementary material

SUPPLEMENTARY FIGURE S1 Removal of batch effects across different datasets. **(A)** Boxplot before batch effect removal shows significant variability in gene expression distributions between the MI and Normal groups across different datasets. **(B)** Boxplot after batch effect removal exhibits the adjusted and normalized gene expression distributions across the MI and Normal groups.

SUPPLEMENTARY FIGURE S2 Functional enrichment analysis of DEGs. **(A)** GO enrichment analysis of DEGs. **(B)** KEGG pathway enrichment analysis of DEG.

## References

[B1] Allen J. L. Hames R. A. Mastroianni N. M. Greenstein A. E. Weed S. A. (2022). Evaluation of the matrix metalloproteinase 9 (MMP9) inhibitor Andecaliximab as an Anti-invasive therapeutic in Head and neck squamous cell carcinoma. Oral Oncol. 132, 106008. 10.1016/j.oraloncology.2022.106008 35803110 PMC13524229

[B2] Bao Y. Wang L. Liu H. Yang J. Yu F. Cui C. (2024). A diagnostic model for Parkinson’s disease based on anoikis-related genes. Mol. Neurobiol. 61 (6), 3641–3656. 10.1007/s12035-023-03753-6 38001358

[B3] Bassiouni W. Ali M. A. M. Schulz R. (2021). Multifunctional intracellular matrix metalloproteinases: implications in disease. FEBS J. 288 (24), 7162–7182. 10.1111/febs.15701 33405316

[B4] Ber Y. Shiloh R. Gilad Y. Degani N. Bialik S. Kimchi A. (2015). DAPK2 is a novel regulator of mTORC1 activity and autophagy. Cell Death Differ. 22 (3), 465–475. 10.1038/cdd.2014.177 25361081 PMC4326577

[B5] Berezin A. E. Berezin A. A. (2020). Adverse cardiac remodelling after acute myocardial infarction: old and new biomarkers. Dis. Markers 2020, 1215802. 10.1155/2020/1215802 32626540 PMC7306098

[B6] Boťanská B. Barteková M. Ferenczyová K. Fogarassyová M. Kindernay L. Barančík M. (2021). Matrix metalloproteinases and their role in mechanisms underlying effects of quercetin on heart function in aged zucker diabetic fatty rats. Int. J. Mol. Sci. 22 (9), 4457. 10.3390/ijms22094457 33923282 PMC8123171

[B7] Bräuninger H. Krüger S. Bacmeister L. Nyström A. Eyerich K. Westermann D. (2023). Matrix metalloproteinases in coronary artery disease and myocardial infarction. Basic Res. Cardiol. 118 (1), 18. 10.1007/s00395-023-00987-2 37160529 PMC10169894

[B8] Capozzi A. Riitano G. Recalchi S. Manganelli V. Costi R. Saccoliti F. (2021). Effect of heparanase inhibitor on tissue factor overexpression in platelets and endothelial cells induced by anti‐β2‐GPI antibodies. J. Thromb. Haemost. 19 (9), 2302–2313. 10.1111/jth.15417 34107171 PMC8456873

[B9] Capozzi A. Manganelli V. Riitano G. Caissutti D. Longo A. Garofalo T. (2023). Advances in the pathophysiology of thrombosis in antiphospholipid syndrome: molecular mechanisms and signaling through lipid rafts. J. Clin. Med. 12 (3), 891. 10.3390/jcm12030891 36769539 PMC9917860

[B10] Chung Y. H. Ortega-Rivera O. A. Volckaert B. A. Jung E. Zhao Z. Steinmetz N. F. (2023). Viral nanoparticle vaccines against S100A9 reduce lung tumor seeding and metastasis. Proc. Natl. Acad. Sci. U. S. A. 120 (43), e2221859120. 10.1073/pnas.2221859120 37844250 PMC10614828

[B11] Dong H. Jia W. Wang C. Teng D. Xu B. Ding X. (2024). Key subdomains of mesencephalic astrocyte-derived neurotrophic factor attenuate myocardial ischemia/reperfusion injury by JAK1/STAT1/NF-κB signaling pathway. Mol. Med. Camb Mass 30 (1), 139. 10.1186/s10020-024-00916-6 39242993 PMC11380330

[B12] Frangogiannis N. G. (2014). The inflammatory response in myocardial injury, repair, and remodelling. Nat. Rev. Cardiol. 11 (5), 255–265. 10.1038/nrcardio.2014.28 24663091 PMC4407144

[B13] Frangogiannis N. G. (2017). The extracellular matrix in myocardial injury, repair, and remodeling. J. Clin. Invest. 127 (5), 1600–1612. 10.1172/JCI87491 28459429 PMC5409799

[B14] Franz S. Ertel A. Engel K. M. Simon J. C. Saalbach A. (2022). Overexpression of S100A9 in obesity impairs macrophage differentiation via TLR4-NFkB-signaling worsening inflammation and wound healing. Theranostics 12 (4), 1659–1682. 10.7150/thno.67174 35198063 PMC8825590

[B15] Gentek R. Hoeffel G. (2017). The innate immune response in myocardial infarction, repair, and regeneration. Adv. Exp. Med. Biol. 1003, 251–272. 10.1007/978-3-319-57613-8_12 28667562

[B16] Goerg J. Sommerfeld M. Greiner B. Lauer D. Seckin Y. Kulikov A. (2021). Low-dose empagliflozin improves systolic heart function after myocardial infarction in rats: regulation of MMP9, NHE1, and SERCA2a. Int. J. Mol. Sci. 22 (11), 5437. 10.3390/ijms22115437 34063987 PMC8196699

[B17] Jin S. Kang P. M. (2024). A systematic review on advances in management of oxidative stress-associated cardiovascular diseases. Antioxidants 13 (8), 923. 10.3390/antiox13080923 39199169 PMC11351257

[B18] Jin J. Zhang J. Bu S. (2022). Tasquinimod efficacy and S100A9 expression in glucose-treated HREC cells. Int. Ophthalmol. 42 (2), 661–676. 10.1007/s10792-021-02038-y 34796432

[B19] Kim C. Kim H. Sim W. S. Jung M. Hong J. Moon S. (2024). Spatiotemporal control of neutrophil fate to tune inflammation and repair for myocardial infarction therapy. Nat. Commun. 15 (1), 8481. 10.1038/s41467-024-52812-6 39353987 PMC11445496

[B20] Krebber M. M. Van Dijk C. G. M. Vernooij R. W. M. Brandt M. M. Emter C. A. Rau C. D. (2020). Matrix metalloproteinases and tissue inhibitors of metalloproteinases in extracellular matrix remodeling during left ventricular diastolic dysfunction and heart failure with preserved ejection fraction: a systematic review and meta-analysis. Int. J. Mol. Sci. 21 (18), 6742. 10.3390/ijms21186742 32937927 PMC7555240

[B21] Li Y. Y. Feldman A. M. (2001). Matrix metalloproteinases in the progression of heart failure: potential therapeutic implications. Drugs 61 (9), 1239–1252. 10.2165/00003495-200161090-00002 11511020

[B22] Li Z. Solomonidis E. G. Meloni M. Taylor R. S. Duffin R. Dobie R. (2019). Single-cell transcriptome analyses reveal novel targets modulating cardiac neovascularization by resident endothelial cells following myocardial infarction. Eur. Heart J. 40 (30), 2507–2520. 10.1093/eurheartj/ehz305 31162546 PMC6685329

[B23] Li C. Weng J. Yang L. Gong H. Liu Z. (2024). Development of an anoikis-related gene signature and prognostic model for predicting the tumor microenvironment and response to immunotherapy in colorectal cancer. Front. Immunol. 15, 1378305. 10.3389/fimmu.2024.1378305 38779664 PMC11109372

[B24] Liu Y. Xu J. Gu R. Li Z. Wang K. Qi Y. (2020). Circulating exosomal miR-144-3p inhibits the mobilization of endothelial progenitor cells post myocardial infarction via regulating the MMP9 pathway. Aging 12 (16), 16294–16303. 10.18632/aging.103651 32843584 PMC7485705

[B25] Liu G. Liao W. Lv X. Zhu M. Long X. Xie J. (2024). Comprehensive analysis of hypoxia-related genes in diagnosis and immune infiltration in acute myocardial infarction: based on bulk and single-cell RNA sequencing data. Front. Mol. Biosci. 11, 1448705. 10.3389/fmolb.2024.1448705 39234566 PMC11371776

[B26] Liu J. Chen X. Zeng L. Zhang L. Wang F. Peng C. (2024). Targeting S100A9 prevents β-Adrenergic activation–induced cardiac injury. Inflammation 47 (2), 789–806. 10.1007/s10753-023-01944-w 38446361

[B27] Ma Y. Yabluchanskiy A. Lindsey M. L. (2013). Neutrophil roles in left ventricular remodeling following myocardial infarction. Fibrogenes. Tissue Repair 6 (1), 11. 10.1186/1755-1536-6-11 PMC3681584 23731794

[B28] Ma X. Zhang L. Gao F. Jia W. Li C. (2023). Salvia miltiorrhiza and Tanshinone IIA reduce endothelial inflammation and atherosclerotic plaque formation through inhibiting COX-2. Biomed. Pharmacother. Biomedecine Pharmacother. 167, 115501. 10.1016/j.biopha.2023.115501 37713995

[B29] Mahtta D. Sudhakar D. Koneru S. Silva G. V. Alam M. Virani S. S. (2020). Targeting inflammation after myocardial infarction. Curr. Cardiol. Rep. 22 (10), 110. 10.1007/s11886-020-01358-2 32770365

[B30] Marinković G. Koenis D. S. de Camp L. Jablonowski R. Graber N. de Waard V. (2020). S100A9 links inflammation and repair in myocardial infarction. Circ. Res. 127 (5), 664–676. 10.1161/CIRCRESAHA.120.315865 32434457

[B31] Martí-Carvajal A. J. Gemmato-Valecillos M. A. Monge Martín D. Dayer M. Alegría-Barrero E. De Sanctis J. B. (2024). Interleukin-receptor antagonist and tumour necrosis factor inhibitors for the primary and secondary prevention of atherosclerotic cardiovascular diseases. Cochrane Database Syst. Rev. 9 (9), CD014741. 10.1002/14651858.CD014741.pub2 39297531 PMC11411914

[B32] Mortezapour M. Tapak L. Bahreini F. Najafi R. Afshar S. (2023). Identification of key genes in colorectal cancer diagnosis by co-expression analysis weighted gene co-expression network analysis. Comput. Biol. Med. 157, 106779. 10.1016/j.compbiomed.2023.106779 36931200

[B33] Mujalli A. Banaganapalli B. Alrayes N. M. Shaik N. A. Elango R. Al-Aama J. Y. (2020). Myocardial infarction biomarker discovery with integrated gene expression, pathways and biological networks analysis. Genomics 112 (6), 5072–5085. 10.1016/j.ygeno.2020.09.004 32920122

[B34] Nandi S. S. Katsurada K. Sharma N. M. Anderson D. R. Mahata S. K. Patel K. P. (2020). MMP9 inhibition increases autophagic flux in chronic heart failure. Am. J. Physiol. Heart Circ. Physiol. 319 (6), H1414–H1437. 10.1152/ajpheart.00032.2020 33064567 PMC7792705

[B35] Nian W. Huang Z. Fu C. (2023). Immune cells drive new immunomodulatory therapies for myocardial infarction: from basic to clinical translation. Front. Immunol. 14, 1097295. 10.3389/fimmu.2023.1097295 36761726 PMC9903069

[B36] Niu L. Geyer P. E. Wewer Albrechtsen N. J. Gluud L. L. Santos A. Doll S. (2019). Plasma proteome profiling discovers novel proteins associated with non-alcoholic fatty liver disease. Mol. Syst. Biol. 15 (3), e8793. 10.15252/msb.20188793 30824564 PMC6396370

[B37] Oliveira J. B. Soares A. A. S. M. Sposito A. C. (2018). Inflammatory response during myocardial infarction. Adv. Clin. Chem. 84, 39–79. 10.1016/bs.acc.2017.12.002 29478516

[B38] Prabhu S. D. Frangogiannis N. G. (2016). The biological basis for cardiac repair after myocardial infarction: from inflammation to fibrosis. Circ. Res. 119 (1), 91–112. 10.1161/CIRCRESAHA.116.303577 27340270 PMC4922528

[B39] Ramos-Regalado L. Alcover S. Badimon L. Vilahur G. (2024). The influence of metabolic risk factors on the inflammatory response triggered by myocardial infarction: bridging pathophysiology to treatment. Cells 13 (13), 1125. 10.3390/cells13131125 38994977 PMC11240659

[B40] Rangel R. Sun Y. Guzman-Rojas L. Ozawa M. G. Sun J. Giordano R. J. (2007). Impaired angiogenesis in aminopeptidase N-null mice. Proc. Natl. Acad. Sci. U. S. A. 104 (11), 4588–4593. 10.1073/pnas.0611653104 17360568 PMC1815469

[B41] Reed G. W. Rossi J. E. Cannon C. p. (2017). Acute myocardial infarction. Lancet Lond Engl. 389 (10065), 197–210. 10.1016/S0140-6736(16)30677-8 27502078

[B42] Rodríguez-Pérez J. M. G V. A. R P. S. Tx Z. J. R OA B. V. A. Valente-Acosta B. (2016). rs3918242 MMP9 gene polymorphism is associated with myocardial infarction in Mexican patients. Genet. Mol. Res. GMR 15 (1), 15017776. 10.4238/gmr.15017776 26985929

[B43] Saberiyan M. Zarei M. Safi A. Movahhed P. Khorasanian R. Adelian S. (2024). The role of DAPK2 as a key regulatory element in various human cancers: a systematic review. Mol. Biol. Rep. 51 (1), 886. 10.1007/s11033-024-09761-6 39105958

[B44] Shabani F. Farasat A. Mahdavi M. Gheibi N. (2018). Calprotectin (S100A8/S100A9): a key protein between inflammation and cancer. Inflamm. Res. Off. J. Eur. Histamine Res. Soc. Al 67 (10), 801–812. 10.1007/s00011-018-1173-4 30083975

[B45] Shen M. Wu Y. Li L. Zhang L. Liu G. Wang R. (2024). CircMAP3K5 promotes cardiomyocyte apoptosis in diabetic cardiomyopathy by regulating miR-22-3p/DAPK2 Axis. J. Diabetes 16 (1), e13471. 10.1111/1753-0407.13471 37735821 PMC10809294

[B46] Shen S. Zhang M. Wang X. Liu Q. Su H. Sun B. (2024). Single-cell RNA sequencing reveals S100a9hi macrophages promote the transition from acute inflammation to fibrotic remodeling after myocardial ischemia‒reperfusion. Theranostics 14 (3), 1241–1259. 10.7150/thno.91180 38323308 PMC10845204

[B47] Shiraishi M. Shintani Y. Shintani Y. Ishida H. Saba R. Yamaguchi A. (2016). Alternatively activated macrophages determine repair of the infarcted adult murine heart. J. Clin. Invest. 126 (6), 2151–2166. 10.1172/JCI85782 27140396 PMC4887176

[B48] Su J. Hu Y. Cheng J. Li Z. Li J. Zheng N. (2023). Comprehensive analysis of the RNA transcriptome expression profiles and construction of the ceRNA network in heart failure patients with sacubitril/valsartan therapeutic heterogeneity after acute myocardial infarction. Eur. J. Pharmacol. 944, 175547. 10.1016/j.ejphar.2023.175547 36708978

[B49] Talley S. Valiauga R. Anderson L. Cannon A. R. Choudhry M. A. Campbell E. M. (2021). DSS-induced inflammation in the colon drives a proinflammatory signature in the brain that is ameliorated by prophylactic treatment with the S100A9 inhibitor paquinimod. J. Neuroinflammation 18 (1), 263. 10.1186/s12974-021-02317-6 34758843 PMC8578918

[B50] Tian Y. Wang Z. Liang F. Wang Y. (2023). Identifying immune cell infiltration and hub genes during the myocardial remodeling process after myocardial infarction. J. Inflamm. Res. 16, 2893–2906. 10.2147/JIR.S416914 37456781 PMC10349602

[B51] Tugal D. Liao X. Jain M. K. (2013). Transcriptional control of macrophage polarization. Arterioscler. Thromb. Vasc. Biol. 33 (6), 1135–1144. 10.1161/ATVBAHA.113.301453 23640482

[B52] Vaswani K. Chan H. W. Verma P. Dekker Nitert M. Peiris H. N. Wood-Bradley R. J. (2015). The rat placental renin-angiotensin system - a gestational gene expression study. Reprod. Biol. Endocrinol. RBE 13, 89. 10.1186/s12958-015-0088-y PMC4532142 26260700

[B53] Vlodavsky I. Barash U. Nguyen H. M. Yang S. M. Ilan N. (2021). Biology of the heparanase-heparan sulfate Axis and its role in disease pathogenesis. Semin. Thromb. Hemost. 47 (3), 240–253. 10.1055/s-0041-1725066 33794549 PMC9097616

[B54] Wang H. Dou L. (2024). Single-cell RNA sequencing reveals hub genes of myocardial infarction-associated endothelial cells. BMC Cardiovasc Disord. 24 (1), 70. 10.1186/s12872-024-03727-z 38267885 PMC10809747

[B55] Wang W. Kang P. M. (2020). Oxidative stress and antioxidant treatments in cardiovascular diseases. Antioxidants 9 (12), 1292. 10.3390/antiox9121292 33348578 PMC7766219

[B56] Wang S. Song R. Wang Z. Jing Z. Wang S. Ma J. (2018). S100A8/A9 in inflammation. Front. Immunol. 9, 1298. 10.3389/fimmu.2018.01298 29942307 PMC6004386

[B57] Wang L. Zhang Y. Yu M. Yuan W. (2022). Identification of hub genes in the remodeling of non-infarcted myocardium following acute myocardial infarction. J. Cardiovasc Dev. Dis. 9 (12), 409. 10.3390/jcdd9120409 36547406 PMC9788553

[B58] Warde-Farley D. Donaldson S. L. Comes O. Zuberi K. Badrawi R. Chao P. (2010). The GeneMANIA prediction server: biological network integration for gene prioritization and predicting gene function. Nucleic Acids Res. 38 (Suppl. l_2), W214–W220. 10.1093/nar/gkq537 20576703 PMC2896186

[B59] Wynn T. A. Vannella K. M. (2016). Macrophages in tissue repair, regeneration, and fibrosis. Immunity 44 (3), 450–462. 10.1016/j.immuni.2016.02.015 26982353 PMC4794754

[B60] Xu Y. Li R. Li X. Dong N. Wu D. Hou L. (2020). An autophagy-related gene signature associated with clinical prognosis and immune microenvironment in gliomas. Front. Oncol. 10, 571189. 10.3389/fonc.2020.571189 33194668 PMC7604433

[B61] Yang X. Xu J. Lan S. Tong Z. Chen K. Liu Z. (2023). Exosomal miR-133a-3p derived from BMSCs alleviates cerebral ischemia-reperfusion injury via targeting DAPK2. Int. J. Nanomedicine 18, 65–78. 10.2147/IJN.S385395 36636640 PMC9830074

[B62] Yang X. Huang Y. Tang D. Yue L. (2024). Identification of key genes associated with acute myocardial infarction using WGCNA and two-sample mendelian randomization study. PloS One 19 (7), e0305532. 10.1371/journal.pone.0305532 39024234 PMC11257238

[B63] Ye Y. Pei L. Ding J. Wu C. Sun C. Liu S. (2019). Effects of Platycodin D on S100A8/A9-induced inflammatory response in murine mammary carcinoma 4T1 cells. Int. Immunopharmacol. 67, 239–247. 10.1016/j.intimp.2018.12.008 30562685

[B64] You G. Zhao X. Liu J. Yao K. Yi X. Chen H. (2023). Machine learning-based identification of CYBB and FCAR as potential neutrophil extracellular trap-related treatment targets in sepsis. Front. Immunol. 14, 1253833. 10.3389/fimmu.2023.1253833 37901228 PMC10613076

[B65] Yuan Y. Huang H. Hu T. Zou C. Qiao Y. Fang M. (2024). Curcumin pretreatment attenuates myocardial ischemia/reperfusion injury by inhibiting ferroptosis, autophagy and apoptosis via HES1. Int. J. Mol. Med. 54 (6), 110. 10.3892/ijmm.2024.5434 39364745 PMC11517743

[B66] Zhang G. Zhang K. (2024). Screening and identification of neutrophil extracellular trap-related diagnostic biomarkers for pediatric sepsis by machine learning. Inflammation 48, 212–222. 10.1007/s10753-024-02059-6 38795170

[B67] Zhang Q. Wang L. Wang S. Cheng H. Xu L. Pei G. (2022). Signaling pathways and targeted therapy for myocardial infarction. Signal Transduct. Target Ther. 7 (1), 78. 10.1038/s41392-022-00925-z 35273164 PMC8913803

[B68] Zhang Y. Wu F. Teng F. Guo S. Li H. (2023). Deficiency of S100A9 alleviates sepsis-induced acute liver injury through regulating AKT-AMPK-dependent mitochondrial energy metabolism. Int. J. Mol. Sci. 24 (3), 2112. 10.3390/ijms24032112 36768433 PMC9916677

[B69] Zhang J. (2022). Biomarkers of endothelial activation and dysfunction in cardiovascular diseases. Rev. Cardiovasc Med. 23 (2), 73. 10.31083/j.rcm2302073 35229564

[B70] Zheng Y. Wang Y. Qi B. Lang Y. Zhang Z. Ma J. (2024). IL6/adiponectin/HMGB1 feedback loop mediates adipocyte and macrophage crosstalk and M2 polarization after myocardial infarction. Front. Immunol. 15, 1368516. 10.3389/fimmu.2024.1368516 38601146 PMC11004445

[B71] Zhi X. Shi S. Li Y. Ma M. Long Y. Li C. (2023). S100a9 inhibits Atg9a transcription and participates in suppression of autophagy in cardiomyocytes induced by β1-adrenoceptor autoantibodies. Cell Mol. Biol. Lett. 28 (1), 74. 10.1186/s11658-023-00486-1 37723445 PMC10506287

[B72] Zhong C. Niu Y. Liu W. Yuan Y. Li K. Shi Y. (2022). S100A9 derived from chemoembolization-induced hypoxia governs mitochondrial function in hepatocellular carcinoma progression. Adv. Sci. Weinh Baden-Wurtt Ger. 9 (30), e2202206. 10.1002/advs.202202206 PMC9596847 36041055

[B73] Zhou X. Zhang W. Jin M. Chen J. Xu W. Kong X. (2017). lncRNA MIAT functions as a competing endogenous RNA to upregulate DAPK2 by sponging miR-22-3p in diabetic cardiomyopathy. Cell Death Dis. 8 (7), e2929. 10.1038/cddis.2017.321 28703801 PMC5550866

[B74] Zhuang L. Zong X. Yang Q. Fan Q. Tao R. (2023). Interleukin-34-NF-κB signaling aggravates myocardial ischemic/reperfusion injury by facilitating macrophage recruitment and polarization. EBioMedicine 95, 104744. 10.1016/j.ebiom.2023.104744 37556943 PMC10433018

