# Supplementary figures and images for: Identification of hub genes in myocardial infarction by bioinformatics and machine learning: insights into inflammation and immune regulation
Source: Front Mol Biosci. 2025 Jun 24;12:1607096. doi: 10.3389/fmolb.2025.1607096 (PMC12234309; doi:10.3389/fmolb.2025.1607096)

(A)

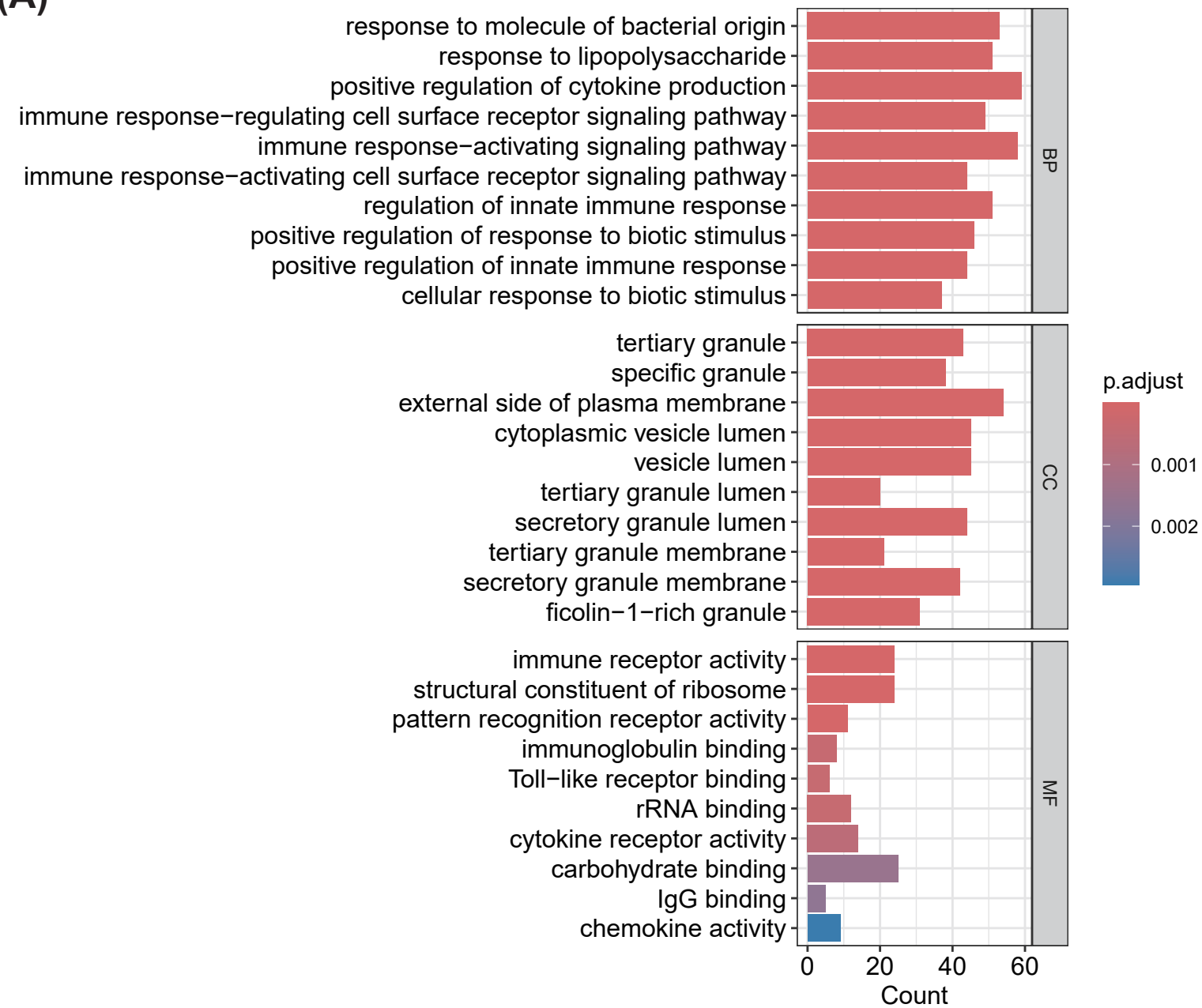

(B)

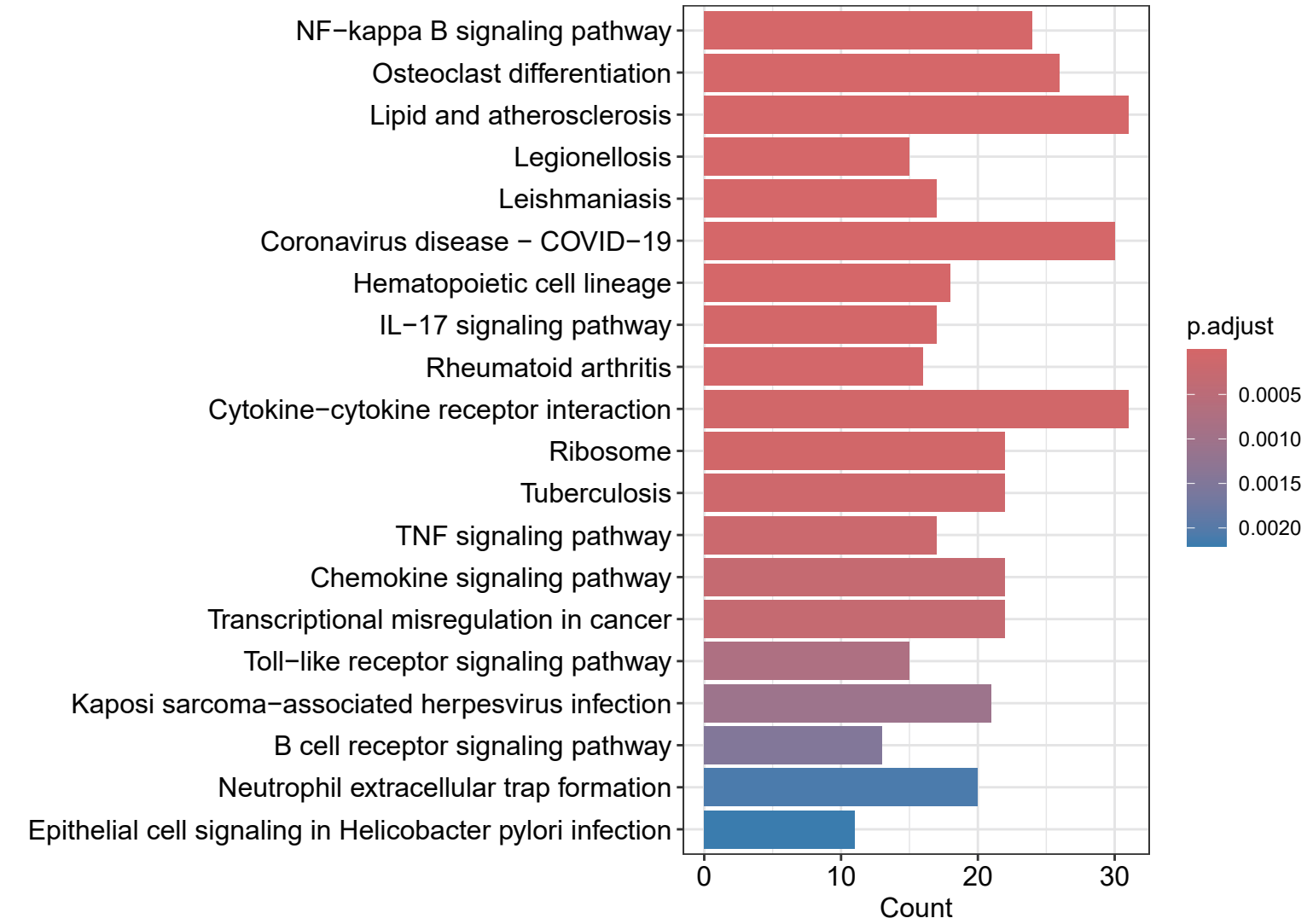

Supplement: Supplementary file 1 [file DataSheet2.pdf]

(A)

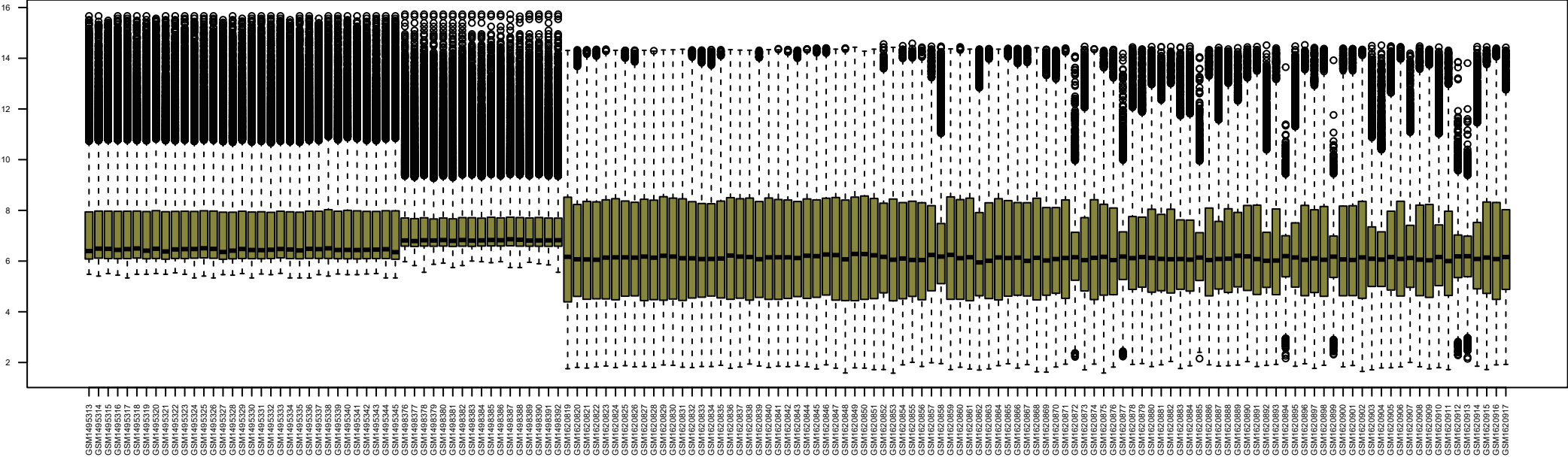

(B)

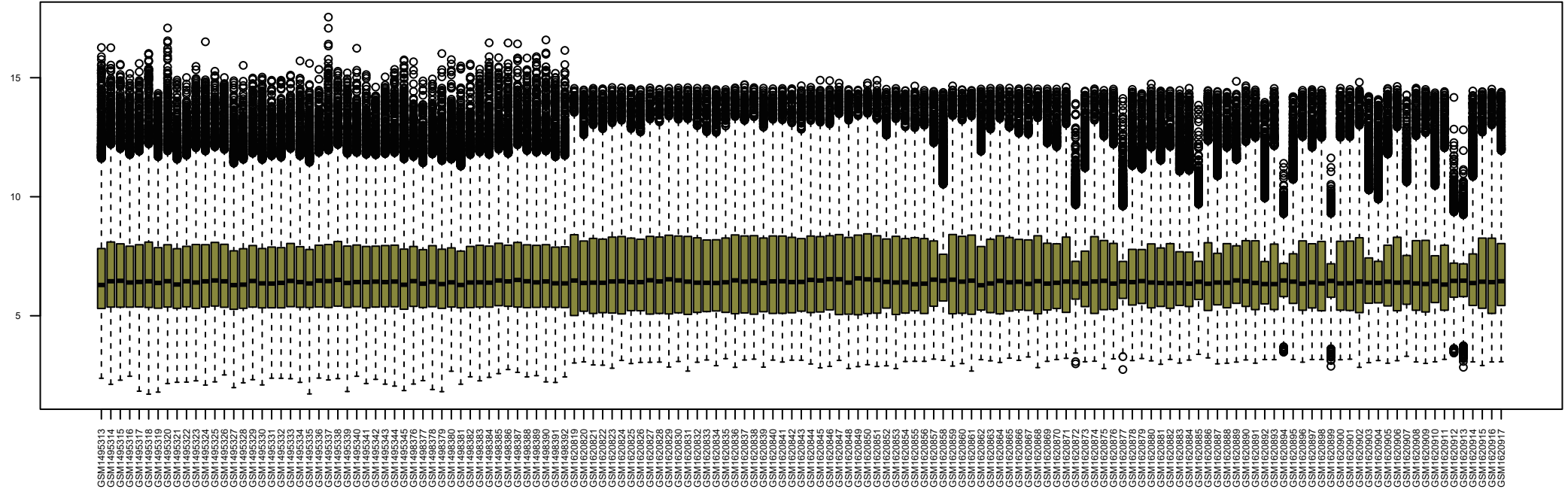

Supplement: Supplementary file 2 [file DataSheet1.pdf]
